# Ethical pinch-points: Effectively communicating ethical consideration in research design

**DOI:** 10.23889/ijpds.v8i2.2316

**Published:** 2023-09-14

**Authors:** Matt Short

**Affiliations:** 1 UK Statistics Authority, Portsmouth, United Kingdom

This presentation aims to communicate the first two year’s work of the UK Statistic Authority’s Centre for Applied Data Ethics (CADE), present key ethical considerations to researchers working with population, administrative and other secondary data, and describe the innovative methods that have been used to review over 700 pieces of research in those two years.

The CADE was established in February 2021 with the aim of enabling researchers and statisticians, both within government and outside of it, to effectively address potential ethical issues in their use of data for the public good. To assist researchers in practically applying data stewardship principles to their research, an ethics self-assessment tool has been developed. The ethics self-assessment process aims to offer researchers an easy-to-use framework to review the ethics of their own projects throughout the research cycle, whilst promoting a culture of “ethics by design”.

In two years’ worth of use, the self-assessment tool has been widely adopted across government, academia and the commercial and charities sectors. Analysis of the work that has been supported by this tool has revealed several topics where researchers could further develop their own research. Well designed and communicated research that demonstrates consideration of these factors enables quicker and safer access to data. In response to these findings, CADE have developed specific guidance pieces to assist the analytical community in considering these ethical concerns in their research design and communicating them effectively to ethics bodies and the wider public to ensure effective and consistent data stewardship practices.

This presentation will support delegates by going into detail in the defining and realising of public good in research, engaging public audiences with research, and considering (and demonstrating) the public view in research design. It will also aid delegates by providing them with a framework that they can use to demonstrate consideration of traditional ethical concerns and also ethical concerns in new, emerging and currently unknown fields.

